# Being a team player: Approaching team coordination in sports in dialog with ecological and praxeological approaches

**DOI:** 10.3389/fpsyg.2022.1026859

**Published:** 2022-12-14

**Authors:** Gerhard Thonhauser

**Affiliations:** Department of Philosophy, Technical University of Darmstadt, Darmstadt, Germany

**Keywords:** team sports, shared affordance, common affordance, collective affordance, collective, synergy, ability-to-play-with

## Abstract

This paper discusses key conceptual resources for an understanding of coordination processes in team sports. It begins by exploring the action guidance provided by the environment, studied in terms of affordances. When conceptualizing sporting performances in general, we might distinguish social and object affordances, think about the spatial and temporal order of affordances in terms of nested and sequential affordances, and differentiate between global, main, and micro-affordances within an action sequence. In the context of team sports, it is crucial to understand how affordances might be given to a plurality of athletes. For that purpose, the paper defines shared, common, and collective affordances. A distinguishing characteristic of team sports is the key role of collaborative intra-team coordination which take place within a setting of antagonistic team-team interactions. A key proposal from dynamical systems theory is to conceptualize intra-team coordination in terms of synergies. Synergies are emergent systems of several athletes who coordinate their movements to achieve specific performance tasks. Many of the embodied skills that players need to develop to become suitable participants in the coordination processes of sport teams are abilities to participate in dynamic sequences of collective activity. Praxeological approaches have emphasized that training processes in team sports are aimed at transforming athletes into skillful participants in sequences of collective play. Athletes need to develop their ability-to-play-with to become proficient in contributing to the formation of suitable collectives for specific performance tasks.

## Introduction

Team sports are about athletes on one team coordinating their actions to form collectives that compete against collectives formed by athletes from the competing team. The key claim of this paper is that we need to approach the coordination processes characteristic of team sports on the level of the team and conceptualize athletes as participants in collective activity. The paper discusses key conceptual issues related to this claim. It does so mostly in dialog with the ecological dynamics approach and the praxeological approach, two major contributors to recent advances in our understanding of coordination processes in team sports. Team coordination in sports introduces the explanandum, arguing that a distinguishing characteristic of team sports is the key role of *collaborative intra-team coordination* which take place within a setting of *antagonistic team-team interactions*. The remainder of the paper identifies key conceptual resources for the study of intra-team and inter-team coordination processes. Following the conceptual orientation of ecological approaches, Types of affordances in team coordination focuses on the role of the environment in the relevant interactions. The aim is to provide a taxonomy of affordances relevant in a team-sporting context. Most importantly, this section distinguishes various ways in which affordances might be given to a plurality of athletes. The issue explored in this section goes beyond a traditional ecological approach that defines affordances individualistically. By contrast, this section argues that athletes not only perceive the affordances of others, but that some affordances can only be realized by a collective of suitably integrated athletes. Teams as synergies explores the integration of athletes into a collective further by building on a key conceptual resource from dynamical systems theory: the notion of a *synergy* which denotes a self-organizing system consisting of at least two athletes. The core idea is that we cannot properly understand the specific dynamics in team sports unless we refer to collectives as agentive centers. This also means that we need to understand the role of athletes primarily as contributors to the activities performed by those collectives. This implies that participation in team sports crucially depends on an athlete’s ability-to-play-with (*Mitspielfähigkeit*), i.e., her ability to skillfully contribute to dynamically unfolding sequences of collective activity. Athletes as skillful participants in collective play turns to this issue, which is a key conceptual resource provided by praxeology. The last few years have seen several proposals for integrated frameworks which might be adopted for future research on team sports. In a brief outlook, I submit that all those frameworks might profit from the conceptual resources discussed in this paper.

## Team coordination in sports

Team sports are highly complex practices that involve multiple dimensions of athlete-environment interactions in need of explanation. This includes the interaction of athletes with material objects (e.g., a ball, puck, or disc, but also the playing surface, and the field boundaries, etc.), a feature team sports share with all sports. A key element of team sports are athlete-athlete interactions. In contrast to material objects, other athletes reciprocally act back which leads to complex dynamics in which athletes mutually affect each other. Some of the athlete-athlete interactions are antagonistic interactions between competing athletes. The paradigmatic example in team sports like basketball or football is the interaction between a dribbling attacker and a defender trying to stop her. This is an aspect which team sports share with other sports, most importantly combat sports. Research on coordination processes in team sports could profit from linking with research on combat sports and other practices that share the nature of *antagonistic athlete-athlete interactions* (Kimmel and Rogler, 2018). But the relevance of antagonistic one-on-one interactions varies from team sport to team sport. For instance, while such interactions are a key element of basketball, they play a much smaller role in volleyball. Finally, there are types of interaction that distinguish team sports from other sports. Team sports are about athletes collaboratively coordinating their movements with the aim of outmaneuvering athletes from an opposing team who aim to do the same. In technical terms, processes of *collaborative intra-team coordination* are crucial, and they take place within a setting of *antagonistic team-team interactions*.

Therefore, if we want to understand the interactions that are specific to team sports, we need to focus on collaborative athlete-athlete interactions in which athletes need to coordination their movements within a dynamically unfolding situation with the goal of outmaneuvering opposing athletes who coordinate their actions with the reciprocal aim of outmaneuvering them. Hence, if we want to understand the interactions that are characteristic of team sports, it is inadequate to deal with the activities of solitary athletes. Team sports are about the skillfulness of intra-team coordination processes. Success in team sports depends on collaborative activities in which the emerging *collective* is the main agentive center. This means that it is ill-conceived to think of team sports in terms of solitary actions by individual athletes who need to be coordinated. A better methodological approach is to understand the actions of athletes as contributions to collective activities. Collectives are nothing above and beyond the athletes, but the structure that appropriately relates athletes so that their contributions mesh into a single collective activity. For instance, it is most suitable to say that the collective is running a set play, because the actions of athletes running a set play are only intelligible as contributions to the collective activity.

In this context, it is important to emphasize that many of the embodied skills that players need to develop to become suitable participants in the coordination processes of sport teams are abilities to participate in dynamic sequences of collective activity. In volleyball, for example, a team needs to return the ball to the other side of the field in a collective action sequence that usually involves three touches of the ball. Each of the three crucial steps in the sequence (bump-set-spike) only makes sense against the background of the collective activity of which it is a part. This example also shows another important feature of intra-team coordination in sports: On the one hand, those coordination processes take place against the background of established sets of movement patterns that function as crucial coordination smoothers. As we will discuss in “Athletes as skillful participants in collective play,” praxeologists refer to those patterns as “practices” (Schatzki et al., 2001). Training in sports is about transforming athletes into experts of the relevant “practices” in the specific sporting domain. On the other hand, those coordination processes are highly dynamic and require a large degree of improvisation. Athletes and teams need to constantly adjust to changing situations. Although one might identify in each sport a limited number of key practices that are regularly enacted, each enaction is unique and requires flexible, fine-grained coordination.

Over the last few years, crucial steps have been made toward the conceptualization of intra-team coordination processes. To begin with, ecological approaches to team sports have focused on quickly emerging and dissolving local interactions through which small groups of players aim to achieve a specific performance task (Araújo et al., 2014; Silva et al., 2015; Araújo and Davids, 2016). Recently, it has been suggested that the ecological framework needs to also account for sociocultural and historic aspects that shape those local interactions (Vaughan et al., 2021; *cf.* Van Dijk and Rietveld, 2017). This is the area in which another framework, namely praxeology, displays its specific strength. Praxeological research on team sports has focused on the cultural contexts, knowledge resources, and training processes that serve a double purpose: On the one hand, they facilitate the development of embodied skills that allow players to be adept participants in the collective activities that a specific sporting domain demands; on the other hand, they enable teams to fine-tune their coordination processes to become proficient collective agents able to quickly and sophistically respond to challenges within the particular sporting environment (Brümmer, 2015; Brümmer and Alkemeyer, 2017; Michaeler, 2018). Praxeologists use the German term “*Mitspielfähigkeit*” to denote the bundle of skills that enable an athlete to excel at intra-team coordination processes. *Mitspielfähigkeit* might be translated as “ability to play along,” “ability to engage in collective activity,” or “ability to be a participant.” For the remainder of this paper, it will be rendered as *ability-to-play-with*. The ability-to-play-with is distinct from other skills relevant in team sports. For instance, a player might be excellent at running, jumping, dribbling, and shooting, but under-average in her ability to coordinate her actions with those of her teammates.

## Types of affordances in team coordination

This section focuses on the role of the environment in intra-team and inter-team coordination processes. How the environment guides behavior is usually conceptualized with help of the term “affordance” (Gibson, 2015). This suggests that one way to approach the role of the environment in coordination processes in team sports is to explore how affordances might be given to a plurality of interacting athletes.

To prepare this exploration, let us begin with some general reflections on the sociality of affordances. The reference to sociality is ambiguous here. In a narrower sense, the term *social affordance* might be used to refer to affordances that provide opportunities for social interaction. This narrower use of the term dates back at least to Valenti and Gold (1991), but the core idea can be traced back all the way to Gibson, who speaks of “mutual and reciprocal affordances” to refer to this kind of affordances: “The other animal and the other person provide mutual and reciprocal affordances at extremely high levels of behavioral complexity.” (Gibson, 2015, p. 129). Gibson explains that the specific feature of those affordances is that they provide opportunities for social interaction: “What the other animal affords the observer is not only behavior but also social interaction.” (Gibson, 2015, 36) In recent research, “social affordances” have been defined by Rietveld (2012, p. 208) as “possibilities for social interaction offered by an environment: a friend’s sad face invites comforting behavior, a person waiting for a coffee machine can afford a conversation, and an extended hand affords a handshake.” They stand in contrast to “object affordances” like “a cup that affords grasping.” (Rietveld, 2012, p. 208). Hence, the distinction between social affordances and object affordances depends on *what* provides the affordance. *Social* affordances are provided by other animals and offer opportunities for social interaction, whereas *object* affordances are provided by material objects and offer opportunities for material practices. Often, the social interactions humans engage in require material objects. This is also the case in a sporting context. For instance, a ball and a basket afford shooting for a lone individual (here, only object affordances are in play), another human without further equipment affords playing tag (here, only social affordances are salient), and another human, a ball, and a basket afford playing one on one (here, we have an interplay of social and object affordances). As the examples show, however, this distinction is rather superficial given the complexity of actual human comportment.

More interesting for our purpose is the sociality of affordances in the wider sense. Rietveld (2012, p. 208) claims that the “responsiveness to object affordances normally partakes within socio-cultural practices.” For that reason, “the responsiveness to object affordances has a normative dimension.” (Rietveld, 2012, p. 209). Together with colleagues, Rietveld has developed this approach to the sociality of affordances into the “Skilled Intentionality Framework” (Bruineberg and Rietveld, 2014; Van Dijk and Rietveld, 2017). They trace their core idea back to Gibson’s introduction of the term “affordance,” but also diverge from it at a crucial step. According to Gibson’s original definition, “the *affordances* of the environment are what it *offers* the animal, what it *provides* or *furnishes*, either for good or ill” (Gibson, 2015, p. 119). Gibson continues to state that affordances “have to be measured *relative to the animal*” (Gibson, 2015, p. 120). Affordances constitute the “niche” of a specific animal and are thus related to the specific “way of life” of that animal (Gibson, 2015, p. 120). In contrast to Gibson’s reference to fixed eco niches of different animal species, Van Dijk and Rietveld (2017) focus on the situatedness of affordances in a sociocultural field within the human form of life. Focusing on what the environment affords to humans, they claim that “we need to understand the human eco-niche as being *sociomaterial* through and through.”^1^ (Van Dijk and Rietveld, 2017, p. 2) In sports, it usually requires years of training to become an expert with a nuanced perception of the relevant affordances and the necessary skills to adequately respond to them. Moreover, sports are rule-based games which means that all affordances within a sporting domain depend upon the rules and conventions of the game. For instance, a ball approaching the ground only affords bumping because of the rules of volleyball (the ball touching the ground within the court being a point, catching being prohibited, etc.). Within the rules and conventions of basketball, by comparison, a ball with the same trajectory might afford catching it after it has bounced off the ground.

Let us review some additional conceptual distinctions in the literature on affordances relevant for the conceptualization of interactions in team sports. To begin with, we can distinguish between *affordances*, denoting “a possibility for action provided by the environment,” and *solicitations*, denoting “an affordance that stands out as relevant [for an athlete] in a specific situation” (Bruineberg and Rietveld, 2014, p. 2). A ball in one’s hand provides an affordance to throw, but whether it also solicits the act of throwing depends on how an athlete orients herself in the situation. Accordingly, we can distinguish between a *landscape of affordances*, which refer to the “whole spectrum of abilities available in our socio-cultural practices” and a *field of affordances*, referring to “the affordances that stand out as relevant for a particular individual in a particular situation.” (Bruineberg and Rietveld, 2014, p. 2). Furthermore, affordances are related to each other over time, with acting on one affordance revealing a subsequent affordance. For instance, catching a ball might lead an athlete to perceive the affordance to throw it. Gaver (1991) coined the term *sequential affordances* to refer to the order of affordances over time. Similarly, affordances are also grouped in space, something Gaver (1991) referred to as *nested affordances*. In fast-paced sports, “many affordances are transient; they evolve and devolve again” (Kimmel and Rogler, 2018, p. 197). A gap in the defense might only be there for a split-second, but the defensive adjustment to close the gap will provide new opportunities for the offense to exploit. In sum, interactions in team sports are characterized by cascades of nested affordances, something team sports share with other antagonistic practices like combat sports and collaborative practices like dance (Kimmel, 2012, 2015; Kimmel and Rogler, 2018).^2^

In contrast to the distinctions made so far, which refer to how one athlete interacts with affordances, the following categories refer to how an *affordance might be given to a plurality of athletes*. To begin with, it has been shown that *shared affordances* allow to explain emergent coordination in various sports (Silva et al., 2013; Kimmel, 2015; Kimmel and Rogler, 2018). For instance, a dribbling attacker and a defender trying to stop her share a cascade of affordances which coordinate their interaction. Such an antagonistic interaction is not only about perceiving the best affordances for oneself and acting on them in the most skillful way, but also about recognizing which affordances the other perceives and using that to one’s advantage. For instance, an athlete might use a fake or feint providing a misleading affordance to her competitor. A fake affordance is a social affordance that is provided by the movements of one athlete (deliberately or as a by-product of her responses to the flow of nested and sequential affordances) and misdirects another athlete. Depending on how the competitor responds to the fake affordance, this provides new affordances to continue the interaction. However, shared affordances are not only relevant in antagonistic but also in collaborative interactions. For instance, a potential receiver needs to see the gap provided to the passer to position herself in the right spot to receive the pass. So, perceiving the affordances of teammates and opponents is a key element of team sports. And more than that, whether the task is to coordinate effectively with teammates or to deceive opponents, athletes also perceive which affordances others perceive them to perceive. For instance, because an athlete sees that her teammate sees her seeing the gap, she passes the ball through the gap because the sharing of the affordance enables her to anticipate that her teammate will move into that gap to receive the pass. In sum, the sharing of affordances refers to how the affordances available to one athlete are disclosed to others. The ability to perceive the affordances available to others is crucial both in antagonistic and collaborative interactions.

Second, Knoblich et al. (2011, p. 63) coined the term *common affordance* to refer to cases in which two agents who are familiar with the same sociomaterial practice and thus share a landscape of affordances perceive the same object which offers them the same type of affordance. This way of conceiving the matter appears rather misleading, as it is highly unlikely that the same object provides several athletes with the same affordance. Usually, an object (for instance the ball, puck, or disc) provides different players with different affordances, depending on their positioning on the field, their role on the team, the specific game situation, and many other factors. However, what is important to consider is that coordination processes are often facilitated by environmental scaffolds (Høffding and Satne, 2021). Athletes do not always need to coordinate their movements through direct interaction because they can rely on each other responding to changes in the environment in a skillful way based on shared practices. In such instances, athletes do not need to perceive each other to coordinate their movement, because they instead use a scaffold provided by the environment. For instance, the movement of the ball or the ball carrier in sports like basketball and football provides affordances to players to adjust their positioning. A player putting pressure on the ball might not be able to see what her teammates are doing because they are positioned behind her back. Nevertheless, she will approach the ball carrier in a specific way aligned with her anticipation of how her teammates will skillfully respond to the affordance provided to them by the movement of the ball. In such cases, the movement of the ball serves as an environmental scaffold facilitating the collective repositioning of the team. Hence, in contrast to the way in which Knoblich et al. defined the term, I submit to use the term *common affordances* in cases in which an object in the environment provides several athletes with different but structurally coordinated affordances which facilitate coordinated movement.

Third, there are affordances for two or more individuals which would not be an affordance for each of them solitarily. Knoblich et al. (2011, p. 63) use the example of a two-handled saw, which only affords cutting for two agents working together, but not for a solitary individual. Another example is moving an object which is so heavy or large that it does not afford lifting for one human of average strength, but only for a sufficiently large group. In the team-sporting context, an example for such an activity is passing. It is important to note that passing is a collective activity that necessarily involves two distinct contributions: sending and receiving a pass. One might independently exercise the physical skills involved in passing (e.g., throwing and catching in the case of sports like basketball or handball). However, the collective activity of passing cannot be reduced to the sum of throwing and catching. The affordance to pass is a collective affordance that is irreducible to the affordances to throw and to catch. On the contrary, within a team-sporting context, affordances to throw and catch are only intelligible as opportunities to contribute to the collective activity of passing. These are challenging cases for traditional ecological approaches. In all these cases, it is impossible to realize the affordance for one individual, as the realization of the affordance is contingent upon the co-action of others. As ecologists stress that affordances are in action, they might be worried about affordances that require contributions of others for their realization. However, this worry might be seen as the manifestation of an individualistic bias that needs to be overcome for the ecological framework to become a viable contributor to the explanation of collective activities (Weichold and Thonhauser, 2020; Thonhauser and Weichold, 2021).

Weichold and Thonhauser (2020) introduced the term *collective affordance* to refer to affordances the subject of which is not a solitary athlete, but a collective of suitably coordinated athletes. We understand a *collective* as a “dynamically constituted and ecologically situated perception-action system that emerges whenever two or more appropriately subjectivized organisms dynamically interact with each other against the background of their relevant embodied social identities” (Weichold and Thonhauser, 2020, p. 2). Anticipating the discussion of synergies in the next section, it is important to note that not all synergies are collectives in our sense. First, the organisms forming the synergy must be capable of having embodied social identities, which roughly refer to their self-understanding in action. It is an empirical question which organism have this ability, but for the purpose of this paper, we can safely assume that athletes competing in team sports possess it. Second, there is some vagueness regarding the threshold of integration required for a collective to emerge, which concerns a complex mix of conceptual and empirical issues. But the gist of the proposal is this: Some synergies are constituted in such a way that they form a new agentive center, and we suggest calling those synergies collectives. Combing this notion of collectives as agentive centers with the subject-dependency of affordances––affordances are relative to the “effectivities” (Turvey and Shaw, 1979) or “acceptances” (Weichold, 2018) of a specific subject––We claim that some affordances are ontologically dependent on collectives. This means that they do not exist without several athletes being integrated in a way that enables them to act as a unit.

This provides a neat explanation of our examples: The affordance to pass can only be realized by a collective consisting of at least two athletes, one sending and one receiving the pass. Similarly, using a two-handled saw is only an affordance for a collective of two individuals doing their part in a coordinated effort. However, when interpreting these examples with an individualistic bias, they might appear ambiguous. We might interpret them collectively, in which case it is straightforward to say that they, as a collective, are passing. But we might also interpret them individualistically, such that it is a case of each individual using the other as a means to her individual ends (Weichold and Thonhauser, 2020, p. 18). Two athletes playing catch might be an instance of each of them using the other to exercise their throwing and catching abilities, or it might be an instance of them together, as a collective, performing a pass. Research on team sports has shown that individualistic assumptions in the cultural background are an important factor impeding collaborative intra-team coordination processes (Michaeler, 2018; Rasmussen et al., 2019; Vaughan et al., 2022), and thus, the emergence of collectives. On the other hand, the same research has shown that forming and maintaining collectives is a major concern of practitioners in team sports and, accordingly, one of the main goals in training design.

Collectives (like synergies) are structures that integrate individual contributions into collective activities, and they can be perceived by observers like coaches who analyze the game. However, being part of a collective is also something that participating athletes perceive. For instance, skillful athletes perceive whether a situation affords a quick counterattack, and this is an affordance that is not perceived as an affordance for me, but *for us.* Ontologically, the affordance to perform a counterattack (usually) does not exist for a solitary athlete, but only for a collective of athletes outnumbering the opposition. In terms of experience, this affordance is perceived as an action opportunity for us. And if the collective performs the counterattack, this is accompanied by a sense of plural agency, a sense of *us* doing it (Schmid, 2016; Satne, 2020).

## Teams as synergies

To repeat the core claim of this paper: If we want to understand the specific dynamics in team sports, *teams* need to be understood as agentive centers. This section reviews a proposal provided by the ecological dynamics framework which is helpful in further cashing-out this claim. The proposal is to conceptualize interacting players in terms of *synergies*. The ecological dynamics framework combines ecological psychology––with its focus on the action opportunities provided by the environment––with dynamical systems theory––focusing on dynamical self-organization in system-environment interactions (Davids and Araújo, 2010).

Dynamical systems theory is built around the notion of *dynamical self-organization*, which means that a system constantly re-organizes its internal structure while interacting with its environment (Vallacher et al., 2002). This implies that a higher-level system, for instance, a group of players who dynamically coordinate their actions, is more than the sum of its parts. There are emergent properties of self-organization that need to be observed on the higher level. Moreover, a focus on dynamical self-organization underscores that the way in which a system behaves is not fixed in advance, but rather emerges and evolves dynamically in its interaction with the environment. As Araújo and Davids (2016, p. 2) summarize, “a dynamical systems approach to sport performance describes how patterns of coordinated movement come about (‘emerge’), persist, and change.”

A key notion of the dynamical systems approach to team coordination is that of a *synergy*. This notion traces back to Bernstein (1967) who understands a synergy as a task-specific coupling of components in which the degrees of freedom of the behaviors of the components mutually regulate each other. Turvey (2007, p. 659) provided the following influential definition, according to which a synergy is “a collection of relatively independent degrees of freedom that behave as a single functional unit – meaning that the internal degrees of freedom take care of themselves, adjusting to their mutual fluctuations and to the fluctuations of the external force field, and do so in a way that preserves the functional integrity of the collection.” In a nutshell, a synergy achieves a reduction of complexity through the reciprocal regulation of degrees of freedom. In the context of team sports, Araújo et al. (2014) and Araújo and Davids (2016), summarizing the work of others, identified four properties of synergies: First, dimensional compression, which refers to the already discussed reduction of degrees of freedom of the synergy in comparison to its components. Second, reciprocal compensation, meaning that if one component contributes differently than expected, the other components adjust their behavior so the collective goal can still be achieved. Third, interpersonal linkages or sharing patterns, which refers to the division of labor between the components. Forth, degeneracy, which indicates the interchangeability of components and the adaptability of their interaction patterns. Dynamical self-organization implies that the way in which a synergy responds to the affordances provided by its environment is sometimes stable and at other times flexible, depending on what is required to achieve the target outcome in shifting contexts.

It is important to emphasize that “a synergy is a functional concept, not a structural, component-based concept” (Araújo and Davids, 2016, p. 5). This means that synergies are task-specific and transient; they are “formed and dissolved rapidly” (Silva et al., 2015, p. 39). Synergies quickly emerge and dissolve as the dynamics of the game provide constantly shifting opportunities for collective activity. This also implies that a synergy does not need to involve an entire team. It depends on the specific sporting domain and the specific situation who is part of the coordination process that leads to the emergence of a synergy. In some sports like volleyball, it is more likely that the entire team forms a synergy. In other sports, it is more reasonable to assume that a team most of the time consists of several smaller synergies. For instance, a defensive line in American Football might be divided into smaller synergies formed by the defensive linemen and the defensive backs, with tight coordination within these groups and only loose coordination among the entire team. However, this is just a hypothesis for conceptual clarification which would need to be empirically tested. When researching synergies empirically, one should be careful not to ignore or dismiss peripheral or minor contributions. To take an example from basketball, a shooter standing in the corner might be a crucial contributor to the attacking synergy. For without her occupying one defender, there would not be space for the play to evolve. In virtue of standing in the right spot (supposedly removed from ‘the action’) instead of attempting to contribute closer to the ball, she most effectively contributes to the collective performance.

The key strength of the concept of “synergy” is that it allows to zoom in on the coordination processes of a small number of players who engage in thick and fast-paced interactions to achieve a specific performance goal. However, researchers working within the ecological framework have also emphasized that factors “like the players’ individual characteristics, a nation’s traditions in a sport, strategy, coaches’ instructions, etc., may impact on the functional and goal-directed synergies formed by the players to shape a particular performance behavior.” (Silva et al., 2013, p. 769) But within an ecological dynamics approach, those factors have traditionally been conceptualized as constraints that are external to the analyzed coordination process. Hence, we can conclude that the way in which team coordination is conceptualized within the ecological dynamics approach––i.e., as narrowly localized both spatially and temporally––shows its strength in the analysis of coordination processes as they dynamically unfold but is less well-equipped to account for all those factors that form the broader context within which those local interactions emerge. By contrast, praxeological approaches to team sports focus on the processes through which players and teams are enculturated into a specific way of doing a sociomaterial practice and how this shapes the way in which they perform within that practice (Brümmer and Alkemeyer, 2017), while paying less attention to the actual mechanisms through which teams achieve fine-grained coordination. Hence, the ecological dynamics approach and the praxeological approach have complementary strengths and weaknesses which makes them promising partners within an integrated transdisciplinary framework.

## Athletes as skillful participants in collective play

This section reviews some key conceptual contributions from praxeology. The key methodological assumption of praxeology––also called practice theory––is taking *practices* at the center of our understanding of social processes (Schatzki, 1996; Schatzki et al., 2001; Reckwitz, 2003). One prominent suggestion is to understand practices as “open, temporally unfolding nexuses of actions” (Schatzki, 2002, p. 72). Practices are action patterns that become habitualized through repeated performance, which means that the relevant actions can easily be repeated in the future. Practices precede specific actions and prefigure the action opportunities within a domain by setting criteria for what are appropriate moves. To be able to act within a practice, agents need to learn the specific moves of that practice. This also implies that agents do not precede practices, but rather emerge within them. This is why praxeologists prefer to speak of *participants*, as this emphasizes that one needs to acquire the appropriate skills to become a potential participant in a practice (Schatzki, 1996, 2002). An example of a practice in football may be playing the ball with two touches, controlling the ball with the first and passing it to a teammate with the second touch. One needs to acquire the skills required to control the ball with the first touch and be ready to pass with the second to become capable of playing with two touches. Acting according to a practice is no automatism though. Although it is the same pattern of two touches, each iteration is different and requires the athlete to dynamically adjust her movement to the specific situation as it unfolds. However, the established practice is crucial in orienting participants about what the appropriate moves are. Experienced players and coaches will correct novices according to their understanding of the practice. But that does not mean that practices themselves are rigid and stable. Practices also change over time as new participants are introduced or as established participants modify their way of doing.

Praxeological research on team sports has focused on the training processes through which athletes are transformed into skillful participants in specific practices, with ethnographic studies exploring training processes in acrobatics (Brümmer, 2015; Brümmer and Alkemeyer, 2017), volleyball (Michaeler, 2018), and football (Brümmer, 2018, 2019). A key finding is that training processes can be seen from two perspectives which are two sides of the same coin: On the one side, training processes in team sports are about skill development through which athletes acquire the abilities required for participation in the relevant practices of the sporting domain. On the other side, training processes are about teams acquiring the necessary coordination patterns to act as a unit. Those two sides are inseparable parts of each training process. Only analytically, they can be distinguished by the theorist.

Another key finding is that many moves of athletes within a team-sporting environment are constitutively parts of dynamically evolving collective activities. The movements of athletes only become intelligible when interpreted as contributions to the collective activity that the relevant collective aims to achieve. This implies that the quality of an athlete’s performance within a team-sporting environment can only be evaluated against the background of the performance goals of the collective task to which it is meant to contribute. Michaeler (2018) has shown how the movements which athletes exercise in volleyball are all organized around the key pattern of bump, set, and spike. Volleyball might be a particularly neat example to make this point, because it demands a high degree of intra-team coordination within a relatively inflexible setting in which antagonistic athlete-athlete interactions play only a minor role. But the same can also be said about other team sports. Taking the example of passing again, which is a key practice in sports like basketball, handball, and football. An athlete might be an excellent thrower and catcher when considering those techniques in isolation, but not good at passing. There are several reasons for this. To begin with, the skills involved in throwing and catching would be ill-conceived from the start if we take them out of the context of passing. For instance, whether a pass is good or bad depends on, among other things, what it enables the receiver to do, i.e., how it contributes to the continuation of collective play. In addition, passing requires constant coordination between the involved athletes (e.g., in how they position themselves in relation to each other, teammates, and members of the opposing team). This shows again that skills in team sports need to be conceived of as abilities to contribute to dynamic sequences of collective activity.

Taking this finding serious has significant consequences for how we think of performance analysis, talent evaluation, skill development, and the design of training sessions. As we have just noted, the performance goals of collective activities are the necessary background for evaluating the quality of athletes’ contributions. This means that performances in team sports need to be analyzed considering their contribution to achieving collective performance goals. Skill development in team sports needs to focus on how the skills of athletes contribute to collective performance tasks of the relevant collective. Therefore, skill training should be organized in a way that not only develops physical skills, but simultaneously develops the athletes’ ability-to-play-with, i.e., their ability to be capable participants in sequences of collective activity. Training designs need to consider that the development of athletes’ abilities is best understood as a process of mutually enabling each other to become better participants. Hence, training settings should aim to facilitate processes that allow for a mutual development of players’ abilities to contribute to collective play.

But not only players, also teams need to develop their coordination patterns through training processes. In this context, it can be noted that it is more common among praxeologists than among ecologists to assign agency to teams. The notion of a synergy discussed in the pervious section is very broad and includes cases of coordination that do not amount to the emergence of a new agentive center. The notion of a *collective* introduced toward the end of “Types of affordances in team coordination” integrates ideas provided by dynamical systems theory and praxeology. It builds on the understanding of dynamical self-organization developed in terms of synergy but combines it with a praxeological view on agency. Praxeologists understand agency as something that is assigned within specific practices: One becomes an agent within a practice by learning the relevant moves and subsequently being treated by others as an agent. Because this definition of agency is ontologically neutral, it neither assumes that all organisms are agents, nor that collectives cannot be agents. Instead, it depends on the specific practice who counts as an agent. Within many practices relevant in the domain of team sports, it is obvious that collectives perform as agents and are treated as such, and for that reason, praxeologists refer to them as collective bodies (Michaeler, 2018).

In team sports, the refinement of coordination patterns that enable collectives to emerge and the habitualization of athletes to become suitable contributors to those collectives usually go hand in hand. Training processes aimed at refining team coordination patterns simultaneously develop athletes’ abilities as participants, and vice versa. Good training design facilitates interactions among players that contribute to both aims at the same time. An example of such a training design is the rondo, a famous exercise in football in which a larger number of players (usually five) aim to maintain possession of the ball by passing to each other, while a lesser number of players (usually two) aim to take the ball from them. Vaughan et al. highlight why this is such a well-designed exercise: “The rondo spotlights passing and receiving opportunities by creating a relevant field of affordances that invite players to embody (i.e., partially realize) the value in teamwork and collaboration. In other words, players must coordinate their movements to create passing affordances and maintain possession.” (Vaughan et al., 2021, p. 8) Similarly, the defending players must move as a unit to avoid opening gaps through which the players in possession of the ball might pass. Hence, the rondo is a prime example of two competing collectives. It is a controlled training setting in which players can refine their ability-to-play-with, both when in possession and when out of possession of the ball. The rondo is meant to focus the attention of players on the task of coordinating with teammates within a sequence of collective activity.

However, collectives are fragile and how successfully they are formed depends not only on the athletes’ abilities-to-play-with, but also on various sociocultural factors in the broader environment. Vaughan et al. have shown through ethnographic work “that a sociocultural value-directedness toward individual competition overshadows opportunities […] for *collective collaboration* in football” (Vaughan et al., 2022, p. 15) and they suggest that coaches should aim to counter that sociocultural trend “by designing training sessions with task constraints that require teamwork and collaboration” (Vaughan et al., 2019, p. 12). Similarly, case studies by Michaeler (2018) and Brümmer (2019) suggest that it is counter-productive if coaches address individual athletes in contexts of collective performances (like in a game situation) because it makes them aware of their own role in intra-team coordination processes which negatively impacts their attunement into the collective activity. By contrast, intra-team coordination runs smoothest if team members are focused on the collective task and act as a unit. All of these highlight the ability-to-play-with as a crucial skill in team-sporting performances that needs to be fostered through training design and through an organizational culture that values teamwork.

## Outlook

This paper has discussed three key conceptual resources for the study of collaborative intra-team coordination in sports. First, it explored how affordances might be given to a plurality of athletes and how they guide collaborative as well as antagonistic interactions in sport. Second, it was discussed how athlete-athlete interactions might lead to the formation of collectives within which athletes coordinate their movements to achieve specific performance tasks. Third, it was shown how training in team sports is about enabling athletes to become skillful participants in sequences of collective activity. Being a good performer in team sports crucially depends on the ability-to-play-with which is one of the factors determining how proficient an athlete is in contributing to the formation of skillful collectives.

These are exciting times for research on team sports. An important issue in current debate is developing an integrated transdisciplinary framework that might be able to combine key findings from different approaches. As an outlook, let me mention three candidates for such an integrated framework: The Skilled Intentionality Framework (Bruineberg and Rietveld, 2014; Van Dijk and Rietveld, 2017), praxeological enactivism (Weichold, 2018; Weichold and Rucińska, 2021), and intercorporeal phenomenology (Meyer and Wedelstaedt, 2017). Exploring these approaches is beyond the scope of this paper. However, I submit that within all those frameworks, an understanding of collectives as agentive centers and the corresponding conceptual resources explored in this paper are important contributions.

## Author contributions

The author confirms being the sole contributor of this work and has approved it for publication.

## Conflict of interest

The author declares that the research was conducted in the absence of any commercial or financial relationships that could be construed as a potential conflict of interest.

## Publisher’s note

All claims expressed in this article are solely those of the authors and do not necessarily represent those of their affiliated organizations, or those of the publisher, the editors and the reviewers. Any product that may be evaluated in this article, or claim that may be made by its manufacturer, is not guaranteed or endorsed by the publisher.

## Footnotes

^1^Van Dijk and Rietveld (2017, p. 4) claim that the relation of affordances and sociocultural practices is one of co-constitution: "It is an example of a constitutive relation because (i) the practice and the affordances that take shape within it are interdependent: any affordance will imply a practice for realizing it and any practice will imply a landscape of available affordances. Furthermore (ii) practices and affordances do not admit of a prioritization."

^2^In addition, Kimmel and Rogler (2018, pp. 204–205) suggest distinguishing between global, main, and micro affordances. An example of a *global affordance* is the task of scoring a basket where athletes may sense how close they are to achieving the goal. A related *main affordance* might be provided by the defenders forward positioned leg, which invites the dribbler to attack this side of her body. *Micro affordances* help to dynamically adjust the movement within the activity of driving past the defender.
